# Maternal resveratrol regulates the growth performance, antioxidant capacity, and intestinal health of suckling piglets through intestinal microorganisms at high summer temperatures

**DOI:** 10.3389/fnut.2022.971496

**Published:** 2022-09-09

**Authors:** Yi Zhao, Yujian Huang, Kaiguo Gao, Xiaolu Wen, Shenglan Hu, Li Wang, Zongyong Jiang, Hao Xiao

**Affiliations:** State Key Laboratory of Livestock and Poultry Breeding, Ministry of Agriculture Key Laboratory of Animal Nutrition and Feed Science in South China, Guangdong Public Laboratory of Animal Breeding and Nutrition, Guangdong Key Laboratory of Animal Breeding and Nutrition, Maoming Branch, Guangdong Laboratory for Lingnan Modern Agriculture, Institute of Animal Science, Guangdong Academy of Agricultural Sciences, Guangzhou, China

**Keywords:** sow, resveratrol, antioxidant, intestinal microbiota, piglets

## Abstract

**Background:**

Resveratrol has numerous beneficial properties, including antioxidant, anti-inflammatory, and immunomodulatory properties. High summer temperatures in Southern China affect the reproductive performance of sows. The present study aimed to investigate the effects of dietary resveratrol supplementation in different thermal environments on the reproductive performance, antioxidant capacity, immune function, and intestinal microbes of sows and piglets during late gestation and lactation, as well as their relationship with colostrum immunoglobulin.

**Methods:**

A two-phase experiment was conducted with 40 healthy multiparous sows. In the first phase of the experiment, 20 sows were used in a moderate temperature environment, and in the second phase of the experiment, the remaining 20 sows were used in a high-temperature environment. In both phases, sows were fed either a control diet or a diet consists of control diet and 300 mg/kg resveratrol starting on day 75 of gestation. Plasma, milk, and fecal samples were collected to obtain the indices of antioxidant capacity, immune function, and intestinal microbes.

**Results:**

The results showed that resveratrol supplementation increased the number of live births by 13.24 and 26.79% in the first and second phases, respectively, compared with the control group. In the second phase, resveratrol supplementation increased litter weight at weaning and in the concentrations of growth hormone (GH), insulin (INS), progesterone (PROG), triglycerides, and uric acid (UA). The plasma superoxide dismutase (SOD) level on day 110 of gestation and day 14 of lactation, as well as glutathione peroxidase (GSH-Px) on day 14 of lactation in the first phase, showed an increasing trend (*p* = 0.0728, *p* = 0.0932, and *p* = 0.067, respectively) in the resveratrol group, compared with the control group. On day 14 of lactation, the plasma total antioxidant capability (T-AOC) level was higher in the second phase, while the plasma malondialdehyde (MDA) level was lower in both phases in the resveratrol group. Resveratrol supplementation increased the abundance of immunoglobulin A (IgA), immunoglobulin G (IgG), and immunoglobulin M (IgM) in colostrum and the relative abundance of *Lactobacillus* and *Alloprevotella* but decreased the relative abundance of *Escherichia-shigella* in piglet feces in the second phase. In addition, Spearman's correlation analysis indicated that the weight gain of weaned piglets was positively (*p* < 0.05) associated with IgM content in colostrum and the abundance of *Lactobacillus* in the fecal microbiota of piglets in the second phase. Moreover, the abundance of *Alloprevotella* was positively correlated with the contents of IgA and IgG in colostrum, while the abundance of *Lactobacillus* was positively correlated with IgM content.

**Conclusion:**

These findings indicated that maternal resveratrol supplementation could enhance the growth performance, antioxidant capacity, and intestinal health of piglets in a high temperature environment, which might be associated with increased immunoglobin secretion from colostrum.

## Introduction

The reproductive performance of sows has seen a remarkable improvement over the last few years. Sows are more sensitive to heat stress. The most suitable temperature for sows is 18–22°C, while high summer temperatures range between 28 and 32°C. Previous studies found that high summer temperatures has a negative impact on the reproductive performance of sows, such as reduced food intake, leading to sow anestrus, prolonged weaning to estrus intervals, low farrowing rates, and reduced milk production (1, 2). Moreover, high summer temperatures also decreased fertility and prolificacy. In the early stages of sow gestation, high summer temperatures could increase embryonic mortality as a consequence of affecting the farrowing rate and litter size (3). High temperatures in late gestation could decrease the number of healthy piglets and the weight of newborn piglets (4). Furthermore, sows in parity one were more sensitive to heat stress (5, 6). The total number of pigs born was decreased by 0.6 pigs for parity one sows when the outside temperature increased from 25 to 30°C, while it was only decreased by 0.2 pigs for parity zero women (7). However, the effects are very limited in sows at different ambient temperatures.

Resveratrol (3,4,5-trihydroxystilbene) is a natural polyphenolic phytoalexin found in many plants, such as grapes, berries, and peanuts. Resveratrol has a wide range of biological effects, including anti-inflammatory, immunomodulatory, antioxidant, and anticancer effects (6, 8–11). Previous studies showed that resveratrol can directly scavenge reactive oxygen species (ROS) (12) and improve the activities of superoxide dismutase (SOD), glutathione peroxidase (GSH-Px), and catalase (CAT), possible mechanism of which was to activate nuclear transcription factors, such as nuclear factor E2-related factor-2 (Nrf2), activator proteins (AP)-1, and Sirtuin 1 (SIRT1), or through enzymatic modifications (13, 14). Studies have been conducted on the beneficial effects of resveratrol in pigs. Maternal resveratrol may improve the immune system and increase the meat quality of offsprings by altering the myofiber characteristics and antioxidant status (15). Dietary resveratrol supplementation in pigs decreased the serum lipid levels (16). In addition, Meng et al. described that supplementation with resveratrol during gestation and lactation improved the reproductive performance of and antioxidant capacity of sows and piglets (17). Therefore, resveratrol is a potential antioxidant in livestock production to improve product performance and economic efficiency. However, it is unclear whether resveratrol supplementation may improve the reproductive performance and intestinal health of sows and piglets subjected to high temperatures. Therefore, we hypothesize that supplementation with resveratrol during gestation and lactation may influence antioxidant capacity, immune function, and intestinal microbes, as well as their relationship with colostrum immunoglobulin in sows and piglets at different ambient temperatures. Moreover, this study contributed to gaining more insights into the effects of dietary supplementation with resveratrol on sows during gestation and lactation at different ambient temperatures.

## Materials and methods

### Animals and experimental design

**Figure 1:**
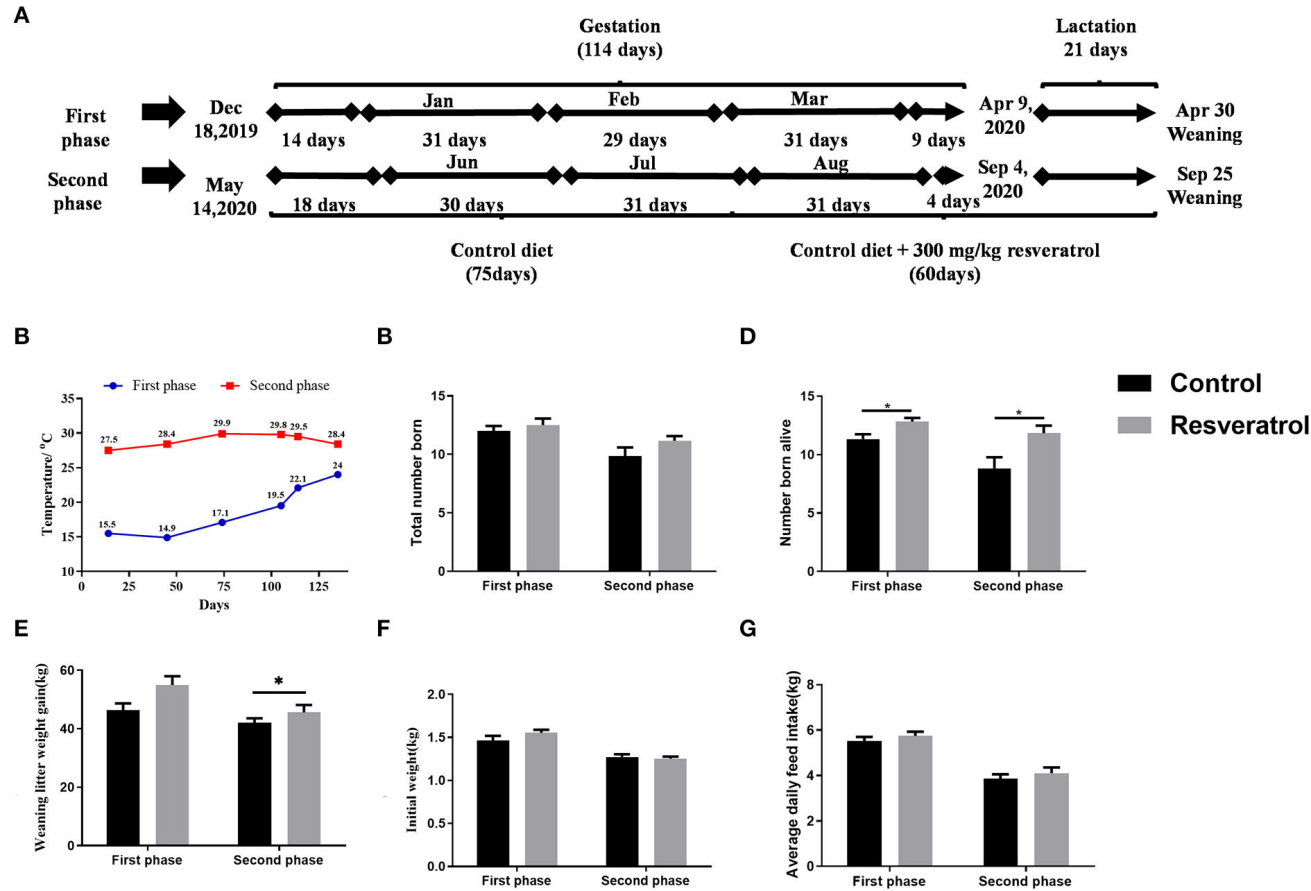
Effects of dietary resveratrol supplementation during gestation and lactation on the reproduction performance of sows. Data were expressed as means ± standard error of the mean (SEM) of at least three independent experiments. **(A)** Schematic diagram of the research design. **(B)** The temperature of the hog house. **(C)** Total number of births. **(D)** Number of live births. **(E)** Litter weight gain at weaning. **(F)** Initial weight. **(G)** Average daily feed intake of sows. *p < 0.05.

The experimental protocol was approved by the Animal Care Committee of the Institute of Animal Science, Guangdong Academy of Agriculture Science, Guangzhou, PR China, under approval number GAASISA-2017-028. In this study, a total of 40 multiparous sows (landrace × large white; three parity) with an initial body backfat thickness of 18.20 ± 1.43 mm (measured at weaning day) were divided into two experimental phases in different seasons. In the first phase, sows were bred in December (environmental temperature was 15–24°C). In the second phase, the sows were bred in May (environment temperature was 27–30°C). Each phase of 20 sows (landrace × Yorkshire, average parity of three) was assigned to two dietary treatments on day 75 of gestation. The treatment groups were as follows: (1) control sows fed a corn-soybean meal control diet (Table 1) (con treatment, *n* = 10) and (2) treatment sows fed a control diet with 300 mg/kg resveratrol (18) (res treatment, *n* = 10). The total period of the experiment was 60 days (from gestation 75 days to lactation 21 days) (Figure 1A). Resveratrol (99% purity) was obtained from Shanghai Aladdin Biochemical Technology Co. Ltd. Sows were kept in individual crates from insemination to day 110 of gestation. On day 110 of gestation, sows were transported and housed in individual farrowing crates. After farrowing, the number of piglets born and the litter size of alive, stillborn, and mummified piglets were all recorded. Individual piglet weights were recorded at farrowing.

**Table 1:** Composition and nutrient levels of the basal diet on (air-dry basis).

**Ingredients**	**Gestation phase**	**Lactation phase**
Corn (8%)	75.85	67.85
Soybean meal (44%)	8.50	23.00
Alfalfa powder (13%)	12.50	–
Fishmeal	–	3.00
Soybean oil	–	2.00
Lysine hydrochloride	–	0.15
CaHPO4	0.80	1.10
Limestone	0.70	1.20
NaCl	0.40	0.45
50% Choline chloride 50%	0.25	0.15
1% Premix^a^	1.00	1.00
Total	100.00	100.00
Nutrient levels*		
DE kcal/kg	3150	3475
NE kcal/kg	2334	2532
Crude protein %	11.17	17.60
NDF %	12.82	8.44
SID Lys	0.38	0.93
SID Met + Cys	0.35	0.52
SID Thr	0.33	0.54
SID Trp	0.10	0.19
TCa	0.62	0.90
TP	0.43	0.63
STTD	0.26	0.41

* Nutrient levels were the calculated values. DE, digestible energy; NE, net energy; CP, crude protein; NDF, neutral detergent fiber.

a The premix provided the following per kg of the diet: VA 10000 IU, VD 1400 IU, VE 40 mg, VK 3.0 mg, VB 10.50 mg, VB12 0.04 mg, nicotinic acid 45 mg, pantothenic acid 20 mg, folic acid 1.2 mg, biotin 0.20 mg, choline chloride 550 mg, Cu 80 mg, Fe 100 mg, Zn 100 mg, Mn 50 mg, I 0.3 mg, and Se 0.25 mg. ^2^SID, standardized ileal digestible.

### Diets and feeding

Corn-soybean meal control diets were formulated according to the nutrient requirements of swine (NRC, 2012). All sows were fed two times a day at 7:30 and 16:30 with a total amount of 2.5 kg/day from day 20 of gestation to day 90 of gestation. All sows were fed 3.0 kg/day from day 91 of gestation until farrowing. On the day of farrowing, sows were offered 2.0 kg of the lactation diet. This amount was increased by 1.0 kg daily until *ad libitum* feeding.

### Sample collection

Within 2 h of farrowing, colostrum was collected on day 7 of lactation, and approximately 40 ml of milk samples were collected from all mammary glands after a 1-ml injection with oxytocin and then frozen at −20°C for further analysis. The plasma from sows was collected on day 105 of gestation and day 14 of lactation from the ear vein. On day 21 of lactation, 14 piglets of similar weight and size (two piglets per litter) in each treatment were selected for plasma collection by venipuncture with heparin-free vacutainer tubes. The blood was centrifuged at 3,000 g for 15 min to obtain the plasma and stored at – 20°C until analysis. On day 21 of lactation, fresh fecal samples from sows and piglets were collected in sterilized tubes from the rectum and stored at – 80°C for further analysis.

### Assessment of immune indices

Commercial enzyme-linked immunosorbent assay (ELISA) kits were used to analyze several parameters in the samples, including the levels of secreted immunoglobulin A (sIgA) (Cusabio, Wuhan, China), immunoglobulin A (IgA), immunoglobulin G (IgG), and immunoglobulin M (IgM) (Mlbio, Shanghai, China) in the plasma and milk.

### Evaluation of antioxidant enzyme activity

The enzyme activities of SOD were determined using commercially available kits (Nanjing Jiancheng Bioengineering Institute, Nanjing, China). First, the buffer and the substrate solution were mixed at a ratio of 1:200 to form the substrate application solution; then, the enzyme solution and the enzyme dilution solution were mixed at a ratio of 1:10 to form the enzyme working solution, which was incubated at 37°C for 20 min and then read at 450 nm.

The enzyme activities of glutathione peroxidase (GSH-Px) and T-AOC in the plasma and milk were determined using commercially available kits (Nanjing Jiancheng Bioengineering Institute, Nanjing, China). Lipid peroxidation in the plasma and milk was determined by measuring malondialdehyde (MDA) using the thiobarbituric acid method and commercially available kits (Nanjing Jiancheng Bioengineering Institute, Nanjing, China).

### Microbial profiling and spearman's correlation analysis

Methods for microbiota profiling and Spearman's correlation analysis were analyzed in accordance with our previous study (19). Briefly, total genomic deoxyribonucleic acid (DNA) from fecal samples was extracted using the E.Z.N.A.^®^ Soil DNA kit (Omega Bio-tek, Norcross, GA, USA) according to the producer's protocols and amplified with a primer specific to the barcode (16S V4–V5 region). Amplicons from 2% agarose gels were extracted and purified with the AxyPrep DNA Gel Extraction kit (Axygen Biosciences, Union City, CA, USA). The purified polymerase chain reaction (PCR) products were quantified by Qubit^®^3.0 (Life Invitrogen) and mixed equally for each of the 24 different amplicon barcodes. The PCR product from 2% agarose gels was extracted and purified using the AxyPrep DNA Gel Extraction kit (Axygen Biosciences, Union City, CA, USA). Sequencing libraries were generated and analyzed using the Illumina genomic DNA library preparation procedure and the Illumina MiSeq platform (Shanghai BIOZERON Co., Ltd.) according to standard protocols. The β-diversity analysis was performed using UniFrac to compare the results of the principal component analysis (PCA) using the community ecology package, R-forge (Vegan 2.0 package was used to generate a PCA figure). Principal coordinate analyses (PCoA) for weighted UniFrac distance and PCA were performed to get principal coordinates and visualize complex multidimensional data. To examine whether microbiota composition was altered by dietary resveratrol supplementation, the relative abundance of major microorganisms (except unidentified species) in the phylum and the genus was analyzed. Microbiota function was predicted using the Phylogenetic Investigation of Communities by Reconstruction of Unobserved States (PICRUSt) based on the Greengene database. The resulting abundance of Kyoto Encyclopedia of Genes and Genomes (KEGG) genes in each group was further categorized into biological pathways. The association of different microbial species with the measured parameters was analyzed by applying Spearman's correlation analysis.

### Statistical analysis

Data are expressed as means ± standard error of the mean (SEM). All statistical analyses were performed using GraphPad Prism 6. Analyses of the two groups were accomplished using an unpaired test with Welch's correction or the Mann–Whitney *U*-test (GraphPad Prism 6). The selection of analysis methods was dependent on whether the data had a Gaussian distribution and the same variance or not. The Gaussian distribution and variance of data were analyzed according to a previous study (20). For the analysis of more than two groups, we adopted the one-way analysis of variance (ANOVA) method. The canonical correlation of litter weight gain at weaning, the colostrum immunoglobulin, and the intestinal microbiota of piglets in a high temperature environment were analyzed using the PEARSON procedure of SPSS (SPSS Inst., Inc., USA), respectively. Differences with the values of *p* < 0.05 were considered statistically significant.

## Results

### Dietary supplementation with resveratrol improved reproductive capacity and affected blood hormones and the biochemical parameters of sows

The results of the reproductive performance of sows are depicted in Figure 1. Dietary resveratrol significantly increased the number of piglets born alive (*p* < 0.05) in both phases (Figure 1D). Dietary resveratrol during gestation and lactation in the second phase increased litter weight gain at weaning (*p* < 0.05). However, dietary resveratrol had no effect on the number of piglet births and piglet birth weight (Figures 1C,F). Dietary resveratrol supplementation during gestation and lactation had no effect on average daily feed intake (ADFI) in both phases (Figure 1G). Therefore, dietary resveratrol supplementation could improve the reproductive performance of sows.

**Figure 2:**
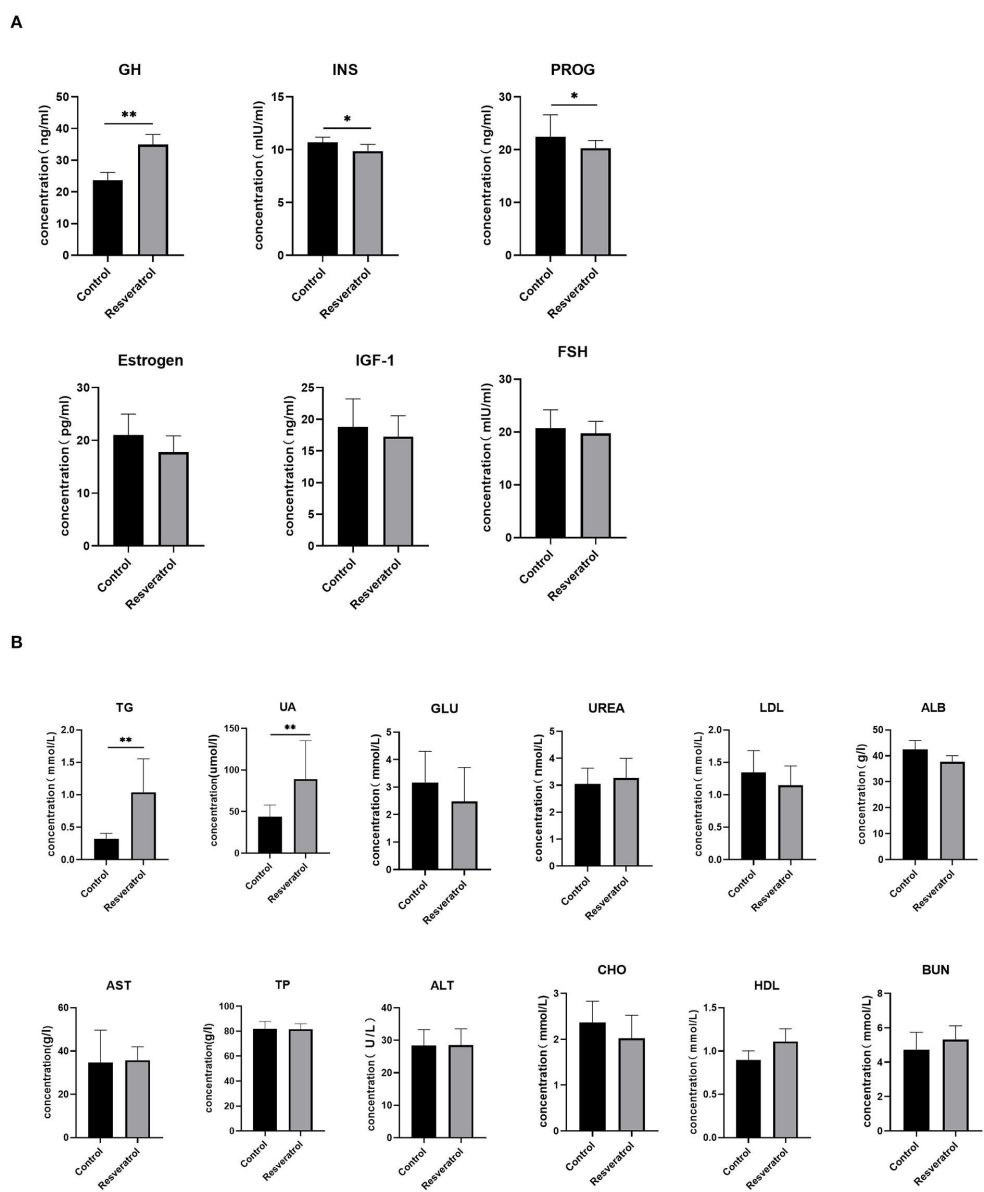
Effect of dietary resveratrol supplementation for the second phase during gestation and lactation on blood hormonal and biochemical markers. **(A)** Blood hormones in sows during 14 days of lactation. **(B)** Biochemical indices of sows during 14 days of lactation. Data were expressed as means ± SEM of at least three independent experiments. GH, growth hormone; INS, insulin; PROG, progesterone; IGF-1, insulin-like growth factor-1; FSH, follicle stimulating hormone; TG, triglycerides; UA, uric acid; GLU, glucose; UREA, urea; LDL, low-density lipoprotein; ALB, albumin; AST, aspartate aminotransferase; TP, total protein; ALT, alanine aminotransferase; CHO, cholesterol; HDL, high-density lipoprotein; BUN, urea nitrogen. *p < 0.05, **p < 0.01.

Resveratrol improved litter weight gain at weaning in the second phase. Therefore, we detected hormones and biochemical parameters in sow plasma during lactation. As seen in Figure 2, dietary resveratrol supplementation increased (*p* < *0.05*) the concentrations of GH, triglycerides (TG), and UA and decreased the secretions of insulin (INS) and progesterone (PROG). However, dietary resveratrol had no significant effect on the concentrations of other blood hormones and blood biochemical parameters (Figures 2A,B).

### Dietary resveratrol supplementation during late pregnancy and lactation affected the antioxidant status in sow plasma, colostrum, and piglet plasma

**Figure 3:**
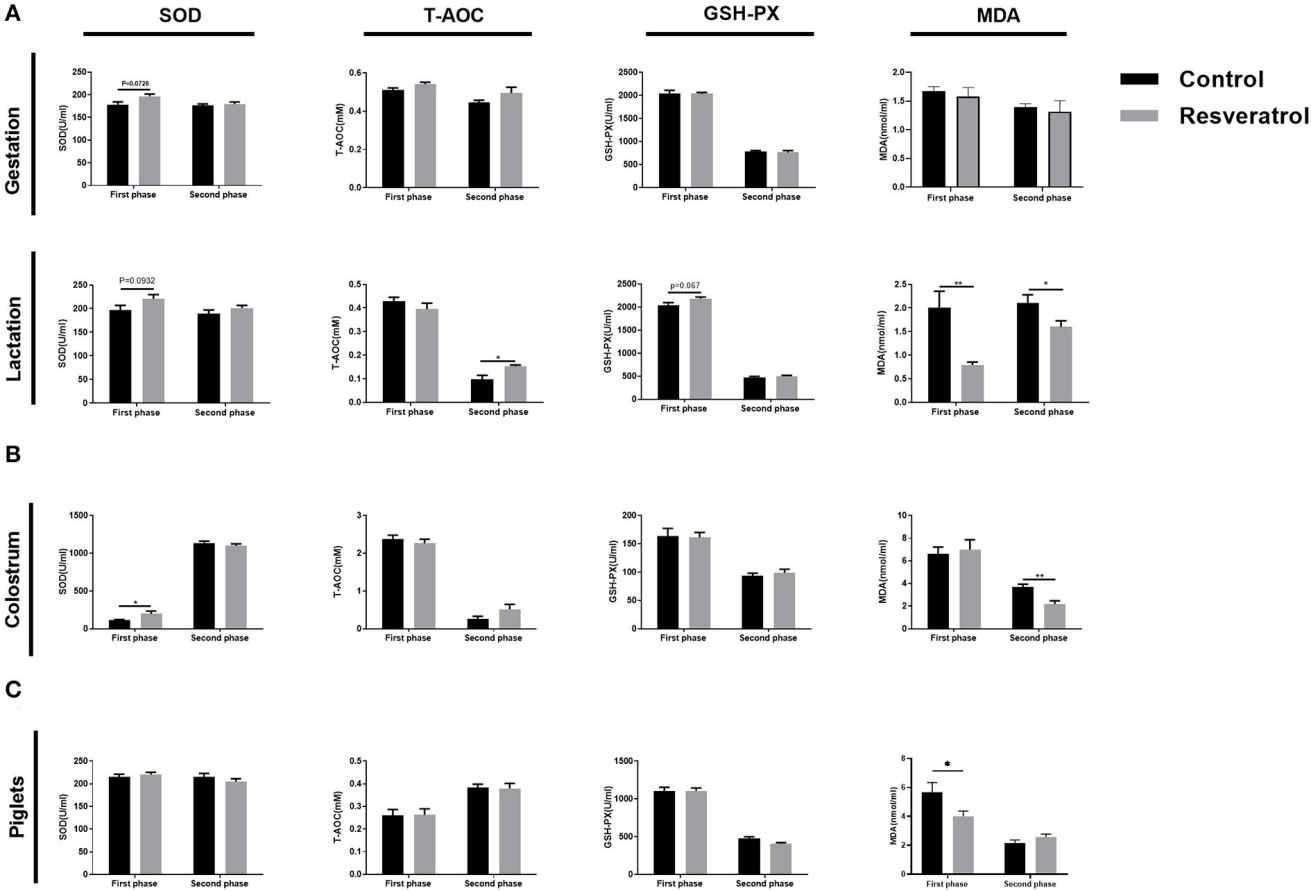
**(A)** Effect of dietary resveratrol supplementation during gestation and lactation on the plasma antioxidant status of sows. **(B)** Effect of dietary resveratrol supplementation during gestation and lactation on the plasma antioxidant status of piglets. **(C)** Effect of dietary resveratrol supplementation during gestation and lactation on the antioxidant status of milk. Data were expressed as means ± SEM of at least three independent experiments. *p < 0.05, **p < 0.01.

The results of the antioxidant status in sow plasma, colostrum, and piglet plasma are shown in Figure 3. During gestation, resveratrol supplementation showed an upward tendency in the activity of SOD in the first phase but had no significant effect on GSH-Px, T-AOC, and MDA levels in sow plasma in both phases. During lactation, dietary supplementation with resveratrol increased (*p* < 0.05) the T-AOC level of sow plasma in the second phase and showed an upward tendency in the SOD and GSH-Px levels in the first phase, while reducing the MDA level of sow plasma in both phases.

As seen in Figure 3B, resveratrol improved (*p* < 0.05) colostrum SOD activity in the first phase and decreased (*p* < 0.05) the colostrum MDA level in the second phase. For piglets, the resveratrol group in the first phase showed a decline in the MDA level (Figure 3C). However, there is no significant effect in other indices in colostrum and piglet plasma between control and resveratrol treatments in both phases (Figures 3B,C). In conclusion, these results suggested that resveratrol improves the antioxidant capacity of sows and transmits it to piglets through lactation.

### Dietary resveratrol supplementation during late pregnancy and lactation increased the immunoglobulin of colostrum in a high temperature environment

**Figure 4:**
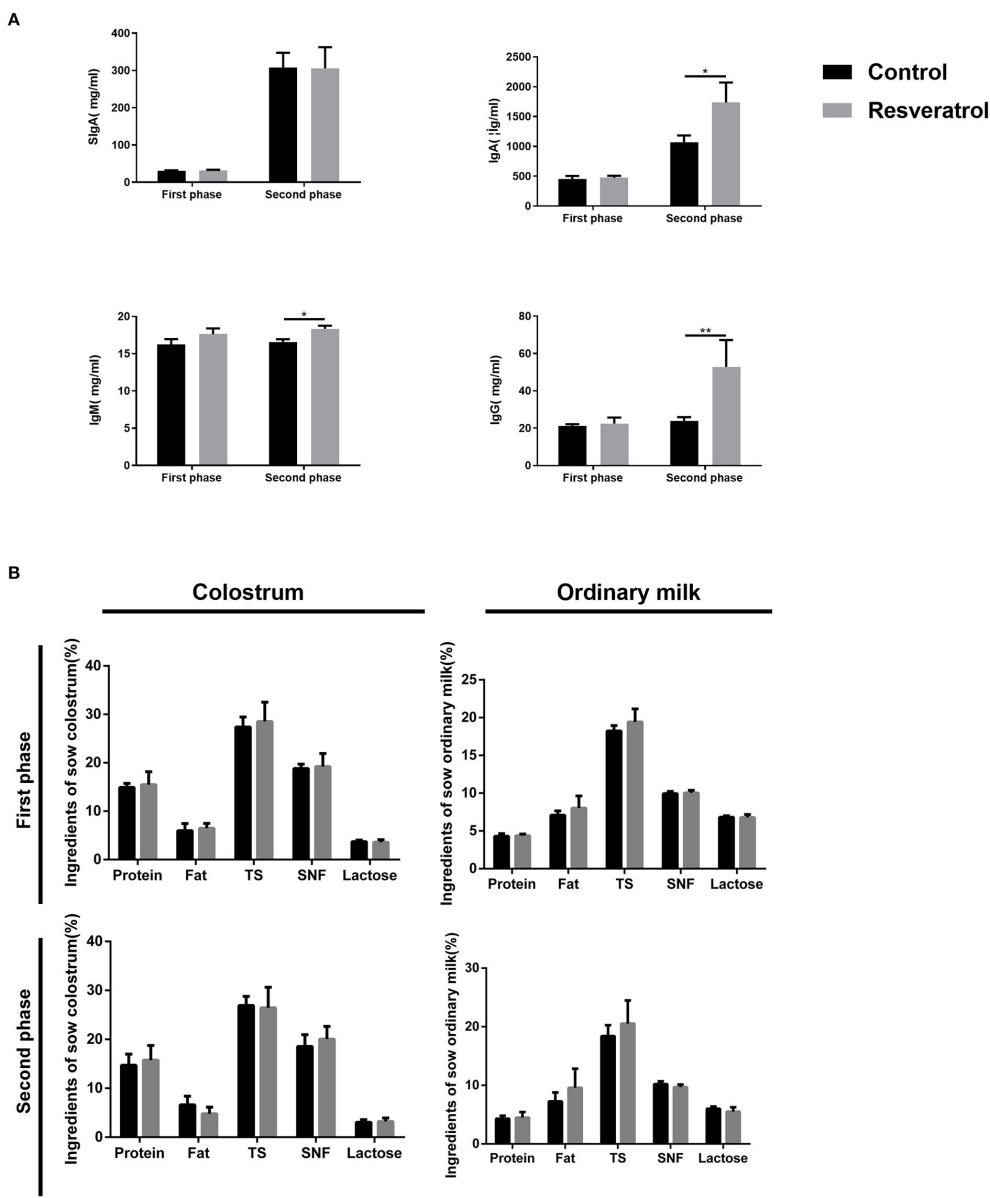
**(A)** Effect of dietary resveratrol supplementation during gestation and lactation on the antioxidant status and immunoglobulin of milk. **(B)** Effect of dietary resveratrol supplementation during gestation and lactation on the composition of colostrum and ordinary milk. Data were expressed as means ± SEM of at least three independent experiments. TS, total solid; SNF, solid non-fat. *p < 0.05, **p < 0.01.

Next, we detected the milk composition and colostrum immunoglobulin. Dietary resveratrol supplementation significantly increased (*p* < 0.05) the IgA, IgG, and IgM contents of colostrum in the second phase, while it had no significant effect on the above indices in the first phase and sIgA in both phases (Figure 4A). As seen in Figure 4B, the composition including the protein, fat, TS, SNF, and lactose (*p* > 0.05) of colostrum and ordinary milk did not differ between control and resveratrol treatments in both phases. Therefore, dietary resveratrol supplementation increased the contents of colostrum immunoglobulin in a high temperature environment.

### Dietary resveratrol supplementation during late pregnancy and lactation transformed the intestinal microbial of piglets in a high temperature environment

**Figure 5:**
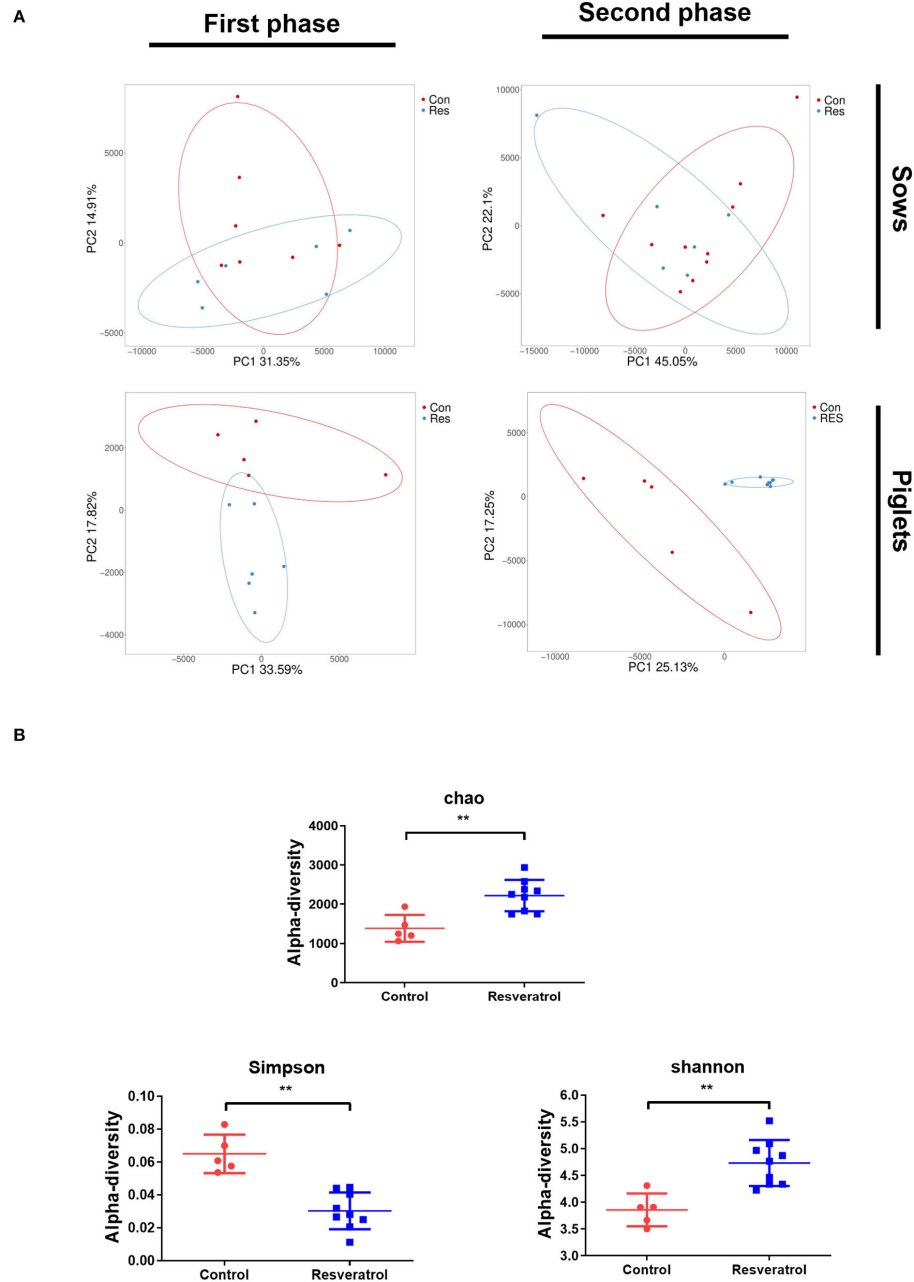
**(A)** Intestinal microbiota composition of sows and piglets by principal component analysis (PCA) analysis. Con, control treatment; Res, resveratrol treatment. **(B)** Intestinal microbiota composition of sows and piglets by α-diversity analysis. Data were expressed as means ± SEM of at least three independent experiments. **p < 0.01.

To examine whether the effect of resveratrol supplementation on piglet weight gain is related to intestinal microbiota, we examined microbiota composition in feces using 16S rDNA amplification sequencing. PCA plots were applied to evaluate differences in β-diversity. PCA plots showed that the microbial community structure of sows and the first-phase piglet samples was not separate between control and resveratrol treatments (Figure 5A). Dots representing the microbial community structure of the second-phase piglet samples was distinctly different in their distribution according to their groups (Figure 5A). The complexity of species diversity was evaluated by diversity indices (Shannon, Simpson and PD whole tree) and richness indices (observed species, Chao1, and ACE) (Figure 5B). Compared to controls, piglets supplemented with maternal resveratrol exhibited a higher richness of microbiota, as demonstrated by increased Shannon (*p* < 0.05), Chao (*p* < 0.05), and Simpon (*p* < 0.05) indices. These results indicated that the microbial structure of piglets was significantly different in the control and resveratrol groups in the second phase.

### Remodeling of the intestinal microbiota of piglets in a high temperature environment was mainly attributed to alterations in *Alloprevotella, Escherichia-Shigella*, and *Lactobacillus*

**Figure 6:**
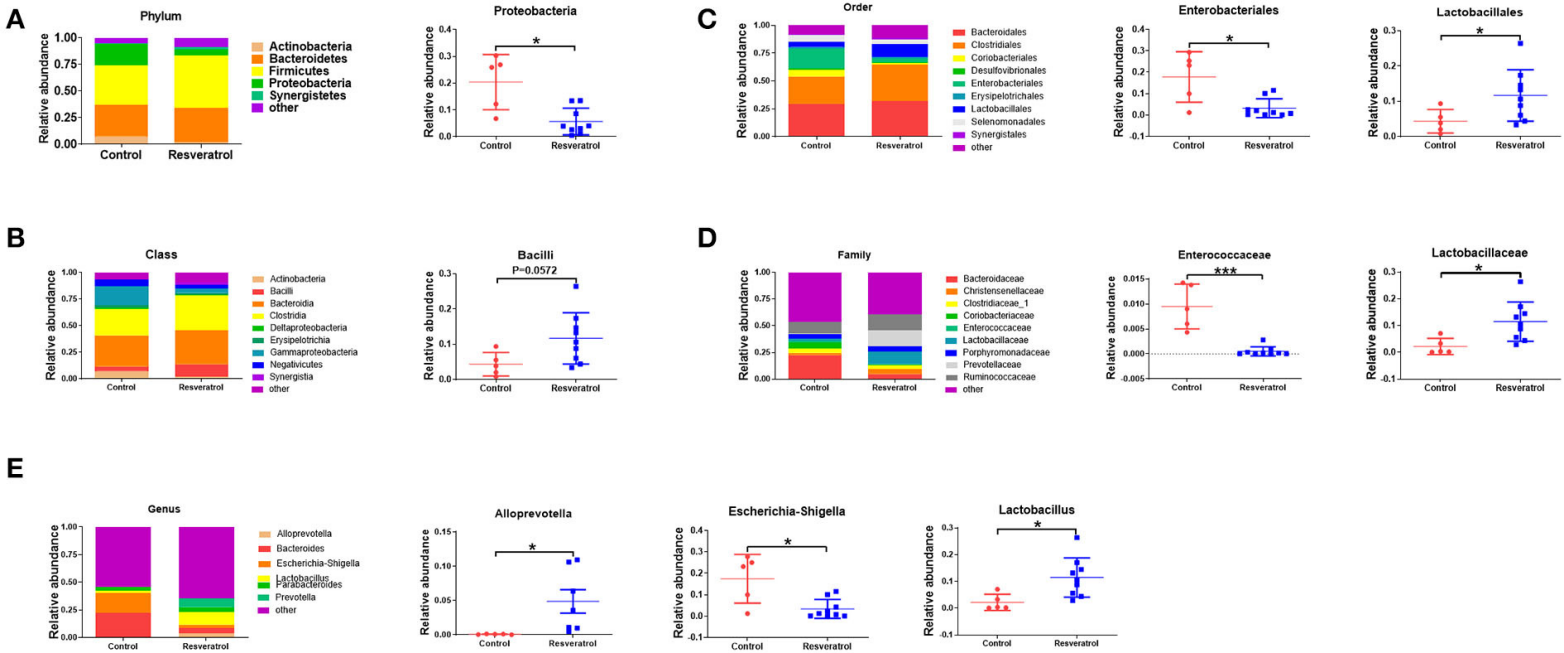
**(A–E)** Relative abundances of main taxa at different levels. Values are means ± SEMs. Differences were assessed by unpaired *t*-test with Welch's correction or Mann–Whitney *U*-test. The selection of analysis methods depends on whether the data were in Gaussian distribution and had equal variance or not. Values are means SEMs. Data were denoted as follows: *p < 0.05, ***p < 0.001.

In the second phase, the largest phylum represented in each group was *Proteobacteria* (Figure 6A). Compared with the control group, the intestinal microbiota of piglets shows a lower relative abundance of *Proteobacteria* (*p* < 0.05) (Figure 6A) and a higher relative abundance of *Bacilli* (*p* = 0.0572) (Figure 6B) after resveratrol supplementation. An increase in the relative abundance of *Bacilli* may ultimately be explained by the augmentation of the relative abundance of *Lactobacillus (p* < 0.05). Accordingly, a decline in the relative abundance of *Proteobacteria* may be attributed to a decline in the relative abundance of *Escherichia-shigella* (*p* < 0.05). In addition, the relative abundance of *Alloprevotella*, a probiotic in the resveratrol group, was higher (*p* < 0.05) compared with the control group (Figures 6C–E). Thus, dietary resveratrol supplementation of sows remodeled the intestinal microbiota and modified the relative abundance of *Lactobacillus, Escherichia-shigella*, and *Alloprevotella* of piglets in a high temperature environment.

### Canonical correlation analysis of litter weight gain at weaning, colostrum immunoglobulin, and the intestinal microbiota of piglets in a high temperature environment

Table 2 shows three canonical relationships between litter weight gain at weaning and colostrum immunoglobulin as well as between litter weight gain at weaning and the intestinal microbiota of piglets and nine canonical relationships between colostrum immunoglobulin and the intestinal microbiota of piglets. The results showed that weaned piglet weight gain was positively (*p* < 0.05) associated with the IgM content in colostrum and the abundance of *Lactobacillus* in the fecal microbiota of piglets in the second phase. Moreover, the abundance of *Alloprevotella* was positively correlated with the contents of IgA and IgG in colostrum, and the abundance of *Lactobacillus* was positively associated with IgM content.

**Table 2:** Canonical correlations between litter weight gain at weaning and immunoglobulin of colostrum with fecal microbiota of piglets.

**The first set of variables**	**The second set of variables**		**Canonical correlation coefficient**	***p*-value**
Weaning litter weight gain	Immunoglobulin of colostrum	IgA	0.172	0.575
		IgG	0.193	0.527
		IgM	0.660	0.014
		Alloprevotella	0.269	0.375
Weaning litter weight gain	Fecal microbiota of piglets	Escherichia-shigella	– 0.122	0.692
		Lactobacillus	0.705	0.007
		Alloprevotella	0.670	0.012
	IgA	Escherichia-shigella	– 0.290	0.336
		Lactobacillus	0.447	0.125
		Alloprevotella	0.694	0.009
	IgG	Escherichia-shigella	– 0.315	0.294
Immunoglobulin of colostrum	Fecal microbiota of piglets	Lactobacillus	0.472	0.104
		Alloprevotella	0.488	0.091
	IgM	Escherichia-shigella	– 0.151	0.622
		Lactobacillus	0.623	0.023

Data were expressed as means ± standard error of the mean (SEM) of at least three independent experiments.

## Discussion

In the present study, we demonstrated that maternal resveratrol improved the growth performance of suckling piglets through intestinal microorganisms at high temperatures. High reproductive sows are more sensitive to heat stress due to increased metabolic heat production. Feed intake is reported to have decreased above 16°C (21). Sows need more energy for milk production during lactation. Therefore, heat stress has a negative effect on sows and piglets. Gilts had fewer piglets born alive and more piglets stillborn due to exposure to high summer temperatures prior to farrowing, which was observed in our study and in previous studies (22, 23). Previous studies showed that resveratrol has become a molecule of potential interest in regulating the reproductive performance of sows (17). However, studies concerning the effects of dietary resveratrol supplementation on sows and piglets in different thermal environments are rather limited. Our results showed that dietary resveratrol supplementation during late pregnancy and lactation for sows had no effect on the total number of births, initial weight, and average daily feed intake in different thermal environments, which are consistent with those of Meng et al. (17). However, we found that maternal resveratrol supplementation increased live births in different thermal environments, as well as litter gain at weaning at high summer temperatures.

Frequent suckling and a negative energy balance are known to be potent stimuli for prolactin and growth hormone (GH) release. The function of GH is to partition metabolites to the mammary gland for milk production and, in negative energy balance, to mobilize body fat and minimize body protein losses (24). Our results showed that dietary resveratrol supplementation prior to farrowing increased the secretions of GH and decreased the secretions of INS and PROG in sows for milk production. Urea (UREA) was determined to exclude massive protein mobilization during lactation. A previous study found that resveratrol supplementation in sows increased the messenger ribonucleic acid (mRNA) level related to lipolysis, fatty acid uptake for piglets from circulating triacylglycerols, and lipogenesis. In this study, we found that maternal resveratrol supplementation improved triglyceride and UA concentrations, which would also contribute to milk production, but further investigation was needed. Sun et al. demonstrated that resveratrol supplementation during gestation and lactation improved colostrum lactose content and milk fat content on day 21 of lactation, which was not observed in our study (25).

Studies reported that a high thermal environment during late gestation may increase the oxidative stress of sows (26). Numerous studies showed that resveratrol can effectively relieve the oxidative stress in pig and rodent models (10, 15, 17, 27, 28). For example, Singh et al. showed that resveratrol prevented embryonic oxidative stress and apoptosis of the diabetic dam (28). Meng et al. demonstrated that dietary resveratrol supplementation during pregnancy and lactation improved the antioxidant status of sows and piglets (17). In this study, resveratrol increased the antioxidant capacity in both sows and piglets, which is consistent with previous studies.

Colostrum intake plays a major role in piglet development by providing energy for thermoregulation, enabling immune transfer from the sows and establishing the gut microbiota (29, 30). In the present study, we found that resveratrol supplementation in the diet of sows increased litter weight gain at weaning, increased the colostrum immunoglobulin (IgA, IgG, and IgM), and transformed intestinal microbial remodeling of piglets during late gestation and lactation in a high thermal environment, which is in agreement with previous research (17). Colostrum immunoglobulin has multiple protective roles in newborns. IgGs absorbed from the small intestine into the circulation provide protection against septicemia and other immunological alterations (31). The results of some studies revealed that the intake of lower concentrations of IgG in late-born piglets is attributed to a decreased survival rate before weaning (32). Therefore, we investigated the relationship between litter weight gain at weaning, the colostrum immunoglobulin, and the intestinal microbiota of piglets in high thermal environments. The results showed that litter weight gain at weaning had a significant canonical correlation with colostrum IgM/lactobacillus. Colostrum immunoglobulin, *Alloprevotella*, and *Lactobacillus* were positively correlated with litter weight gain at weaning, while *Escherichia-shigella* was negatively correlated with litter weight gain at weaning. Previous studies demonstrated that the *Lactobacillaceae* family was regarded as beneficial bacteria for regulating intestinal health by improving intestinal barrier integrity and immunity function (33, 34). Similarly, *Alloprevotella* is closely associated with the production of short-chain fatty acids (34, 35), and *Alloprevotella* mainly produces succinates and acetates, which can improve the intestinal barrier and have an anti-inflammatory function (34, 36, 37). *Lactobacilli* are considered to be potentially beneficial bacteria in the gut, which can prevent the infection or colonization of pathogens by competing for epithelial binding sites and nutrients. It can also inhibit the growth of pathogens by producing active components such as bacteriocins and lactic acid, which will result in the alteration of the ecological balance of intestinal commensal bacteria (38). *Escherichia-shigella* is the most common foodborne epidemical pathogen, which causes diarrhea (39). In summary, maternal resveratrol supplementation can increase litter weight gain at weaning of piglets in a high thermal environment by enhancing colostrum immunoglobulin and improving the probiotics in the intestinal microbiota of piglets.

## Conclusion

In conclusion, the results of the present study suggested that maternal resveratrol supplementation could enhance the growth performance of piglets in a high temperature environment, which might be associated with increased antioxidant status, the secretion of IgA, IgG, and IgM in colostrum, and improved piglet intestinal microbiota. These aspects affect the growth performance of piglets.

## Data availability statement

All the datasets used and analyzed during the current study are included in the manuscript. Availability of data and materials the datasets generated during and/or analyzed during the current study are available the BioProject database, http://www.ncbi.nlm.nih.gov/bioproject/857668 (BioProject ID: PRJNA857668, available on July 11, 2022).

## Ethics statement

The animal study was reviewed and approved by All Animal Experimental Protocols used in this study were according to the Chinese guidelines for animal welfare and approved by the Animal Care and Use Committee of Guangdong Academy of Agricultural Sciences (authorization number GAASIAS-2016-017).

## Author contributions

HX and ZJ conceived the project. YZ and KG performed the sample preparation. YZ, YH, XW, and SH designed the intestinal microbiota analysis process. YZ and YH analyzed intestinal microbiota data, interpreted the data, prepared the original draft of this manuscript. KG visualized the data. YZ, KG, YH, XW, SH, HX, LW, and ZJ revised this paper. All authors contributed to the article and approved the submitted version.

## Funding

This study was jointly supported by the National Natural Science Foundation of China (31902172), the Special Fund for Scientific Innovation Strategy Construction of High-level Academy of Agriculture Science (R2017YJ-YB1004, 202106TD), the Science and Technology Program of Guangzhou City (202005000003), the Project of Swine Innovation Team in Guangdong Modern Agricultural Research System (2022KJ126), and the Start-up Research Project of Maoming Laboratory (2021TDQD002), China.

## Conflict of interest

The authors declare that the research was conducted in the absence of any commercial or financial relationships that could be construed as a potential conflict of interest.

## Publisher's note

All claims expressed in this article are solely those of the authors and do not necessarily represent those of their affiliated organizations, or those of the publisher, the editors and the reviewers. Any product that may be evaluated in this article, or claim that may be made by its manufacturer, is not guaranteed or endorsed by the publisher.

## Abbreviations

ALB: Albumin
ALT: Alanine aminotransferase
AST: Aspartate aminotransferase
BUN: Urea nitrogen
CHO: Cholesterol
FSH: Follicle stimulating hormone
GH: Growth Hormone
GLU: Glucose
GSH-Px: Glutathione peroxidase
HDL: High density lipoprotein
IgA: Immunoglobulin A
IGF-1: Insulin-like growth factor-1
IgG: Immunoglobulin G
IgM: Immunoglobulin M
INS: Insulin
LDL: Low-density lipoprotein
MDA: Malondialdehyde
OTUs: Operational taxonomic units
PCA: Principal component analysis
PROG: Progesterone
SIgA: Secreted immunoglobulin A
SOD: Superoxide dismutase
T-AOC: Total antioxidant capability
TG: Triglycerides
TP: Total protein
UA: Uric acid
UREA: Urea.
